# Next generation sequencing in dilated cardiomyopathy: utility and challenges in the African context

**DOI:** 10.3389/fcvm.2025.1717936

**Published:** 2026-01-19

**Authors:** Minenhle Mayisela, Dineo Mpanya, Umar G. Adamu, Phelelani T. Mpangase, Zané Lombard, Joel Amoni, Nonkanyiso Mboweni, Nqoba Tsabedze

**Affiliations:** 1 Department of Internal Medicine, Faculty of Health Sciences, School of Clinical Medicine, University of the Witwatersrand, Johannesburg, South Africa; 2 Sydney Brenner Institute for Molecular Bioscience (SBIMB), Faculty of Health Sciences, University of the Witwatersrand, Johannesburg, South Africa; 3 Division of Human Genetics, National Health Laboratory Service, Faculty of Health Sciences, School of Pathology, University of the Witwatersrand, Johannesburg, South Africa; 4 Wits Sleep Lab, Faculty of Health Sciences, School of Biomedical Sciences, University of the Witwatersrand, Johannesburg, South Africa

**Keywords:** Africa, dilated cardiomyopathy, genetics, inherited cardiovascular disease, next generation sequencing

## Abstract

Dilated cardiomyopathy (DCM) is a leading cause of heart failure worldwide and has a disproportionately high burden among young Africans. As a primary myocardial disorder, DCM frequently has a genetic aetiology. Although Africa harbours the greatest human genetic diversity, African populations remain underrepresented in genomic research, limiting variant interpretation and clinical applications of next-generation sequencing (NGS). Next Generation Sequencing has transformed the understanding of DCM by enabling comprehensive identification of disease-associated genes. This narrative review summarizes the clinical utility of NGS in the diagnosis, risk stratification, and management of DCM, with a particular focus on Africa. We also highlight some key barriers to implementation, clinical implications, and potential strategies to overcome them. Addressing these challenges through expanded African genomic research, strengthened local capacity, and equitable international collaborations is essential to advance precision cardiovascular medicine and improve outcomes for patients with DCM in Africa.

## 1 Introduction

Dilated cardiomyopathy (DCM) is characterized by left ventricular dilation and regional or global systolic dysfunction that cannot be explained solely by abnormal loading conditions, such as hypertension, valvular disease, or coronary artery disease (1). It is one of the most common forms of cardiomyopathy and is a growing cause of heart failure worldwide (2). The prevalence DCM in SSA according to a systematic review showed it to be a weighted 20.5% (3). Prevalence was shown to vary between regions with 54% in Rwanda and 7.3% in Nigeria (4, 5). Notably, African patients are diagnosed with DCM at a younger age than European patients, with a mean age of 34 vs. 49 years, respectively (6). A recent systematic review reported that in Sub-Saharan Africa (SSA) 21.4% of heart failure is due to cardiomyopathies (7). A twofold increased odds of DCM has been reported among African Americans compared to white individuals (8). The diagnosis traditionally relies on echocardiography, however, cardiovascular magnetic resonance (CMR), which may provide more detailed myocardial tissue characterization, is available for selected cases (9). Next-generation sequencing (NGS) is a powerful tool for filling this knowledge gap, enabling comprehensive genetic evaluation of patients and families. This review explores the potential clinical utility of NGS in understanding DCM genetics in Africa and examines the opportunities and challenges of its implementation in the region.

## 2 Genetics of dilated cardiomyopathy

**Figure 1:**
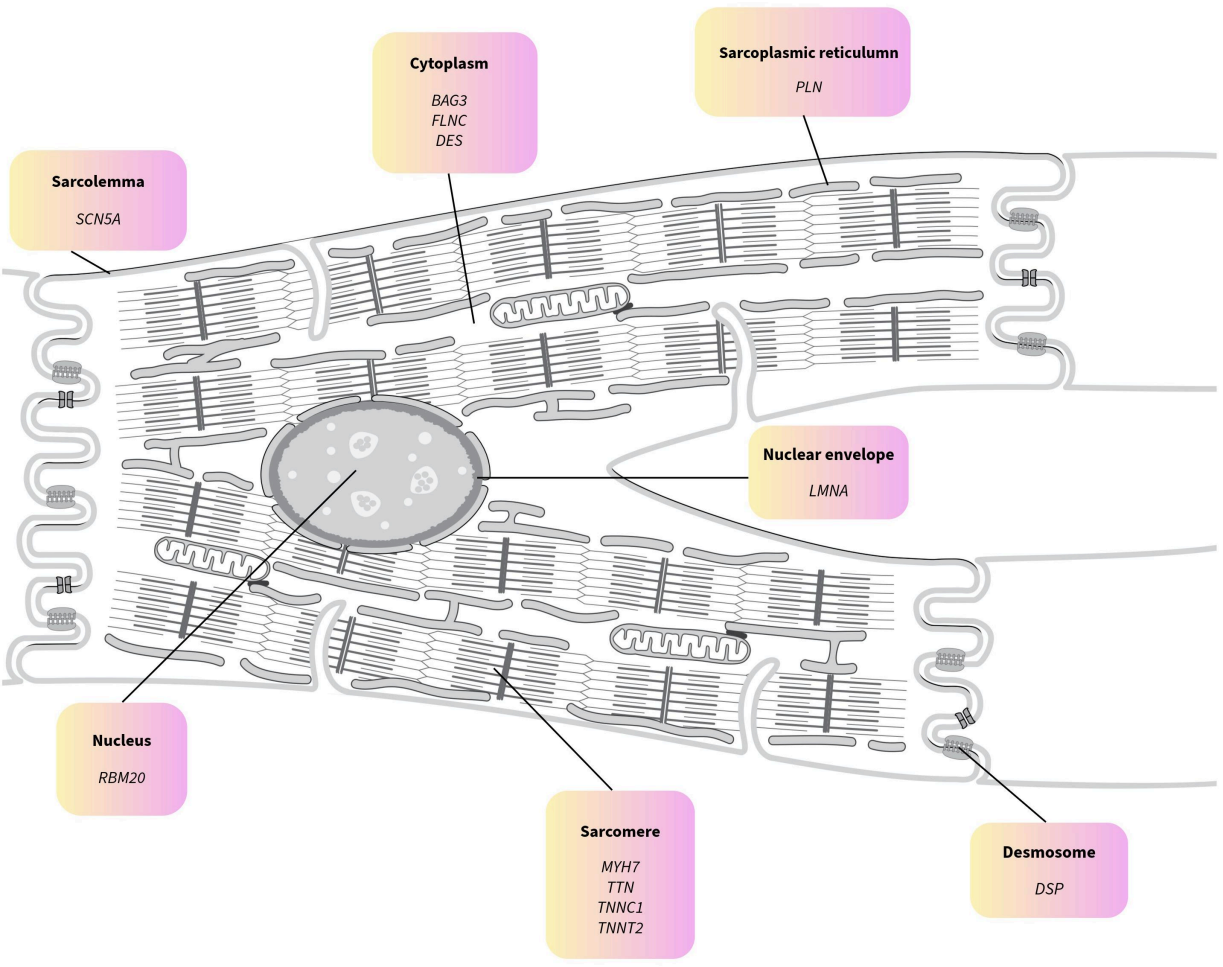
Cellular localization of the 12 genes with definitive association with dilated cardiomyopathy (DCM) as identified by the clinical genome resource. Adapted from “Animal muscle cell” by SwissBioPics, licensed under CC BY 4.0.

DCM is primarily inherited as an autosomal dominant disorder with variable penetrance and expressivity or as a sporadic condition; nonetheless, familial clustering has been observed in 30%–50% of cases (6, 10, 11). Genetically, DCM is highly heterogeneous, with over 200 implicated genes and approximately 60–70 genes supported by clinical evidence, including *TTN, LMNA, MYH7, BAG3, PLN, RBM20*, and *FLNC* (12–14). Of these, 12 have been determined to have a definitive correlation to DCM (13). These genes encode proteins that are critical for cardiomyocyte structure and function, including the sarcomere, cytoskeleton, nuclear envelope, and calcium-handling apparatus. These genes are involved in various structural and functional components. Variants in these genes can impair cardiomyocyte function, leading to ventricular dilation, systolic dysfunction, and heart failure (Figure 1, and Table 1) (13, 15, 16, 20). Titin (*TTN*) truncating variants (*TTNtv*) are the most common genetic cause, accounting for approximately 20%–25% of familial DCM cases, whereas *MYH7* and *LMNA* variants contribute to a smaller but clinically significant proportion (11, 17, 18).

**Table 1:** Genes with definitive association with DCM and their general function (16, 19, 20).

Gene symbol	Gene name	Function
*BAG3*	BAG Cochaperone 3	Promotes autophagy and regulates the HSP70 chaperone system as a nucleotide exchange factor within HSP70 complexes, functioning as an adaptor protein.
*DES*	Desmin	An intermediate filament that links Z-discs, sarcomeres, the sarcolemmal cytoskeleton, the nucleus, and mitochondria, and serves as a sarcomeric microtubule anchor.
*DSP*	Desmoplankin	Anchors intermediate filaments to desmosomes and is essential for desmosomal plaque assembly.
*FLNC*	Filamin C	Binds actin and contributes to the maintenance of sarcomeric stability.
*LMNA*	Lamin A/C	Involved in nuclear lamina and chromatin organization, essential for nuclear dynamics.
*MYH7*	Myosin Heavy Chain 7	Part of the cardiac muscle thick filament, involved in ATPase activity, slow myosin isoform, muscle contraction
*PLN*	Phospholamban	Critical regulator Ca2+ homeostasis, regulates of cardiac ATP2A2 Ca2+ ATPase
*RBM20*	RNA Binding Protein 20	Major regulator of TTN slicing, mRNA splicing regulator of specific target genes
*SCN5A*	Sodium Voltage-Gated Channel Alpha Subunit 5	Facilitates voltage-dependent Na+ permeability of excitable membranes
*TNNC1*	Troponin C1	binds the switch region of troponin I in a Ca2+ dependent manner to activate contraction
*TNNT2*	Troponin T2	Binds troponin complex to tropomyosin
*TTN*	Titin	Major sarcomere scaffolding protein important for assembly, regulation of sarcomere resting length and passive stiffness, protein interaction platform

Emerging genomic studies have identified ancestry-specific genetic contributions to DCM. In particular, a common loss-of-function nonsense variant in *CD36* (rs3211938), which occurs at appreciable frequency in individuals of African ancestry but is nearly absent in European populations, has been associated with a significantly increased risk of DCM and reduced left ventricular function (8). Functionally, this variant impairs myocardial fatty acid uptake and energy metabolism and may contribute to the higher disease burden and earlier onset of DCM observed in African-ancestry populations (8). Despite similar overall variant burdens, pathogenic or likely pathogenic variants are less frequently classified in African cohorts, reflecting the lack of ancestry-specific reference data and limiting their clinical translation.

The phenotypic impact of genetic variation also extends to the modification of clinical severity, age at onset, and sex differences (12, 21, 22). Genetic variations may affect the susceptibility to, and clinical severity of individuals diagnosed with DCM. Genetic variations may also explain age and sex discrepancy in DCM expression (12). Genotype-phenotype correlations, for instance, in *LMNA*, *RBM20*, and *FLNC* carriers, inform arrhythmic risk stratification and prognosis (21, 22). However, the genetic architecture of DCM in African populations remains under characterized, highlighting the urgent need for expanded genomic studies, local biobank development, and inclusion in global cardiogenetic datasets to enable accurate variant interpretation and equitable precision medicine approaches.

## 3 Role of genomic sequencing

DNA sequencing has been available for nearly six decades, with Sanger sequencing long regarded as a highly reliable and widely validated reference method (23). However, the introduction of NGS has further expanded the scope and scale of the field of human genome sequencing. They enable high-throughput, parallel sequencing of millions of DNA fragments simultaneously, which has greatly enhanced the ability to interrogate entire genomes, or large gene sets rapidly and efficiently (24, 25). Next generation sequencing is also associated with a considerably reduced cost, making personalized medicine a modern possibility. In conditions like DCM, where genetic heterogeneity is common, NGS provides a powerful platform for identifying pathogenic variants and advancing personalized care, particularly in underrepresented and low-income populations (25).

**Figure 2:**
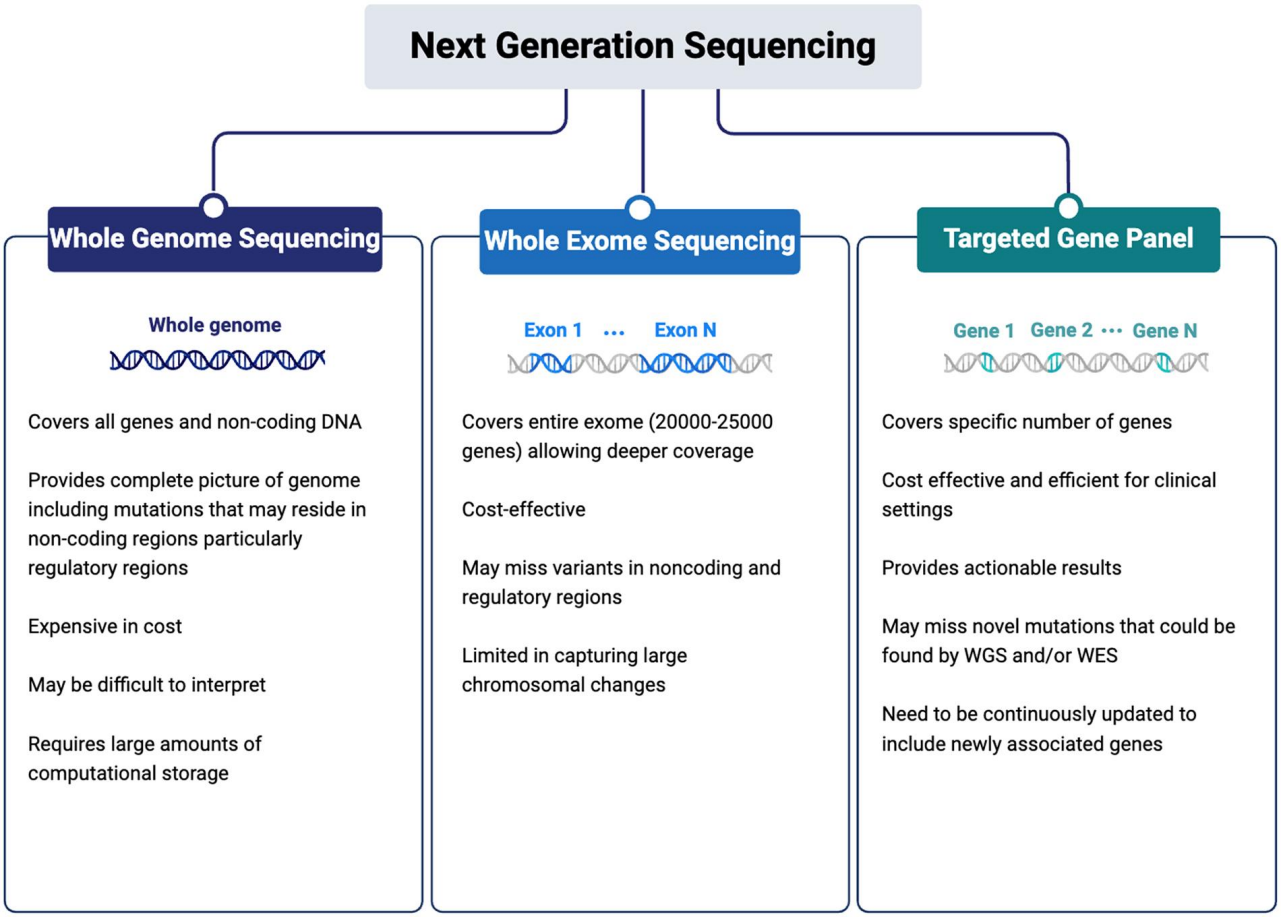
An overview of the methodologies utilized in next-generation sequencing (NGS), including whole-genome sequencing (WGS), whole-exome sequencing (WES), and targeted gene panels (created in https://BioRender.com).

NGS technologies, including whole-genome sequencing (WGS), whole-exome sequencing (WES), and targeted gene panels, offer flexible platforms tailored to different clinical and research needs (Figure 2). WGS provides the most comprehensive assessment by capturing both coding and noncoding regions and detecting structural variants, whereas WES focuses on protein-coding exons that harbour the majority of known pathogenic variants (≈85% of disease-causing variants). Gene panels target curated sets of disease-associated genes and are often used for efficient, cost-effective screening of genetically heterogeneous conditions such as DCM (23–25).

Compared with traditional Sanger methods, NGS significantly reduces costs and turnaround times, enabling genomic testing in larger and more diverse cohorts. The dramatic decrease in sequencing costs over the past two decades, from millions of dollars per genome to under a few thousand, has democratized genomic analysis and accelerated its integration into clinical practice and personalized medicine (25). Moreover, NGS is particularly valuable for genetically heterogeneous disorders such as DCM, where multiple rare variants across many genes contribute to the disease, making comprehensive approaches such as WES and multi-gene panels more effective than single-gene assays. This capacity is especially important for underrepresented and low income populations, where traditional sequencing approaches are impractical and broad variant discovery can fill critical gaps in genomic knowledge (25).

Genomic tools have taken on an essential role in clarifying the genetic factors associated with DCM. Advanced sequencing technologies have led to a rapid increase in the number of genes identified as harbouring variants that are considered causative, primarily through the application of candidate gene approaches (10). NGS has substantially improved our understanding of genotype-phenotype correlations in DCM, allowing for more precise disease characterisation and prognostication (26–28). A meta-analysis of more than 8,000 individuals demonstrated that pathogenic variants in *LMNA*, *PLN*, and *RBM20* are associated with rapid disease progression, conduction abnormalities, and poor outcomes (28). Similarly, *TTN* truncating variants have been shown to correlate with recurrent ventricular arrhythmias and progressive heart failure, independent of variant location within the gene (26). Studies of *MYH7* variants further indicate that DCM associated with this gene is often characterized by early phenotypic expression and frequent left ventricular noncompaction (27).

These genotype-phenotype associations underscore the importance of genetic characterization for risk stratification. A study evaluating genotype-guided prognostic models demonstrated that a genotype-based classification strategy provided superior predictive accuracy for cardiomyopathy-related outcomes compared with phenotype-only approaches (29). In addition, identifying pathogenic variants enables genotype-specific management, including early implantable cardioverter-defibrillator consideration for patients with high-risk genotypes, such as *LMNA* and *DES* variants, which are associated with malignant arrhythmias and sudden cardiac death (30, 31).

Genetic screening also facilitates the identification of asymptomatic carriers of the disease. Studies involving DCM probands and their relatives have reported detection of pathogenic variants in clinically unaffected family members, allowing early surveillance and intervention prior to disease onset (32, 33). This is particularly relevant in families with inherited DCM, as genetic testing improves understanding of disease risk and inheritance patterns (34). Accordingly, cascade testing is recommended for first-degree relatives when a pathogenic or likely pathogenic variant has been identified.

The introduction of NGS into clinical practice has markedly improved the diagnostic yield of DCM. Whole-exome sequencing (WES) achieves diagnostic rates of up to approximately 40% in large cardiomyopathy cohorts, whereas whole-genome sequencing (WGS) may reach yields approaching 70% by capturing non-coding, regulatory, and structural variants not detected by WES (35, 36). In a cohort of 2,088 patients, panel-based testing significantly increased variant detection rates (37). Similarly, a study assessing NGS in paediatric cardiovascular disease reported substantially higher diagnostic yields with NGS, particularly when WGS was employed (38). In Sub-Saharan Africa, the clinical value of NGS has not been fully realised as a diagnostic tool for patients with DCM.

Current guideline-directed medical therapy for DCM, including *β*-blockers, renin-angiotensin system inhibitors, mineralocorticoid receptor antagonists, and sodium glucose co-transporter 2 inhibitors, offers broad benefits but does not account for genotype-specific disease mechanisms (39). This limitation highlights the need for a precise approach. Emerging evidence supports the potential of genotype-guided therapies; for example, lovastatin treatment in patients with *LMNA*-related DCM has been associated with improvements in endothelial dysfunction (40). In parallel, RNA-based therapeutics are gaining momentum in cardiovascular research (41, 42). Preclinical studies using CRISPR-mediated correction of pathogenic *TNNT2* variants have demonstrated reversal of key DCM phenotypes in animal models, highlighting the promise of precision genome editing despite ongoing concerns regarding off-target effects (43).

## 4 African genomic landscape

African populations harbour the greatest human genetic diversity globally but remain markedly underrepresented in genomic research (44–46). This imbalance is particularly concerning for DCM, a condition that is prevalent and poorly characterized across the continent. A recent systematic review identified only a small number of African studies investigating the genetic basis of DCM, of which only one study employed NGS (47). This study reported a pathogenic *LMNA* variant (*LMNA* p.R54C) segregating with disease in affected family members, a variant that has also been associated with DCM-related morbidity and mortality in non-African populations (48). Most genetic studies informing DCM care have been conducted in non-African populations, limiting their clinical applicability to African settings (49). In a large cross-sectional study enrolling patients with DCM from 25 advanced heart failure programs in the United States, significant disparities were observed in pathogenic variant detection and classification between individuals of African and European ancestry, largely driven by differences in reference datasets and variant annotation frameworks (49). The establishment of collaborative initiatives, such as H3Africa, has demonstrated the feasibility and value of large-scale genomic research in Africa (50). Studies arising from these consortia have shown that the inclusion of African genomic data substantially enhances discovery (51, 52). Hence, the reliance on predominantly European genomic data increases the risk of variant misclassification and diagnostic uncertainty in genetically heterogeneous populations, with important implications for clinical decision-making and cascade family screening (53). Given that pathogenic variants inform prognosis, risk stratification, and preventive interventions, a lack of African-specific genomic data may adversely affect patient care.

Investigating African genomes offers a unique opportunity to define the ancestry- and population-specific genetic architectures of DCM. Therefore, the implementation of NGS in African populations is critical for improving diagnostic accuracy, enabling robust genotype–phenotype correlations, and advancing precision cardiovascular medicine on the continent (54, 55).

## 5 Barriers to implementing NGS in the African clinical setting

Despite the growing recognition of NGS as a transformative tool in the diagnosis and management of dilated cardiomyopathy (DCM), its clinical implementation in Africa remains limited by substantial structural, economic, and ethical challenges. These barriers collectively constrain the integration of genomic medicine into routine cardiovascular care (Table 2).

**Table 2:** Barriers to the clinical implementation of next-generation sequencing for dilated cardiomyopathy in Africa and potential solutions.

Barriers	Clinical Implications	Solutions
Limited genomic infrastructure	Few accredited sequencing laboratories, marked regional disparities; reliance on external facilities delays diagnosis and increases costs	Establish regional genomic hubs; integrate NGS within tertiary cardiology and heart failure centres and strengthen public-private partnerships
High cost of sequencing and analysis	Limits access in public funded healthcare systems and restricts routine clinical use	Implement tiered testing strategies (targeted gene panels followed by WES/WGS); pooled procurement; hybrid research-clinical funding models
Shortage of trained personnel	Limited availability of genetic counsellors, medical geneticists, and bioinformaticians impairs interpretation and clinical translation	Capacity-building initiatives; regional training programs; incorporation of cardiovascular genomics into cardiology training curricula
Underrepresentation in genomic reference databases	High rates of variant misclassification and variants of uncertain significance (VUS) and reducing diagnostic confidence	Expansion of African genomic reference datasets thus contributing to global databases; Establish African-led sequencing initiatives
Ethical and informed consent challenges	Low genetic literacy and historical mistrust hinder participation and uptake of testing	Community engagement frameworks; culturally adapted consent processes; development of genetics education tools
Limited access to cascade family screening	Missed opportunities for early diagnosis and preventive interventions in at-risk relatives	Integration of family-based screening into cardiology clinics; task-shifting to trained non-physician healthcare providers
Competing healthcare priorities	Genomic medicine often deprioritized in resource-constrained settings	Demonstration of clinical utility and cost-effectiveness; alignment of NGS with early diagnosis and prevention strategies

A major limitation is the uneven distribution of genomic infrastructure across continents. Although sequencing platforms have been established in parts of Western, Northern, Eastern, and Southern Africa, most notably in South Africa, many regions, particularly Central Africa, remain underserved (56). The scarcity of accredited local genomic laboratories necessitates reliance on external facilities, leading to delays in testing, increased costs, and reduced patient accessibility. Furthermore, the financial burden associated with sequencing, bioinformatics analysis, data storage, and interpretation remains prohibitive for many public healthcare systems, where competing healthcare priorities necessitate careful evaluation of cost-effectiveness (57).

The shortage of trained personnel further impedes the clinical application of NGS. There is a limited workforce of genetic counsellors, medical geneticists, and bioinformaticians capable of interpreting genomic data and delivering appropriate pre- and post-test counselling (58–60). Strengthening local training programs and integrating cardiovascular genomics into existing clinical and academic curricula are essential for ensuring sustainable and context-appropriate genetic-service delivery.

Ethical, legal, and sociocultural considerations also present additional challenges. Informed consent for genomic testing can be difficult in settings with low genetic literacy, language barriers, and limited access to culturally adapted educational resources (61, 62). These challenges may result in an inadequate understanding of testing implications, raising concerns regarding autonomy and the potential misuse of genetic data, particularly in regions with weak regulatory frameworks. Moreover, sociocultural perceptions shaped by historical discrimination and mistrust may further reduce participation in genetic studies and clinical testing. The return of results also poses difficulties, as African populations tend to have a higher burden of variants of uncertain significance (VUS), complicating clinical interpretation and patient communication.

A critical scientific limitation is the underrepresentation of African populations in global genomic databases (45, 63). Reliance on predominantly European-derived datasets leads to higher rates of variant misclassifications and diagnostic uncertainty in individuals of African ancestry. Studies have demonstrated that the use of non-African reference data contributes to genetic misdiagnoses and inappropriate variant classification, underscoring the urgent need for inclusive genomic resources that reflect African genetic diversity (64). Expanding African genomic datasets is likely to facilitate variant reclassification and improve diagnostic accuracy in both affected individuals and asymptomatic carriers.

Addressing these barriers will require coordinated efforts to expand sequencing infrastructure, reduce costs, build human capacity, and establish robust ethical and regulatory frameworks. Importantly, increasing the representation of African populations in genomic research is essential to unlock the full clinical potential of NGS in DCM. Strategic investment in African-led genomic initiatives will enhance risk stratification, prognosis, and family screening and promote equitable access to precision cardiovascular medicine across the continent.

## 6 Future directions and equitable solutions for Africa

Although there are challenges in implementing NGS in the management of DCM in Africa, there are also opportunities. The establishment of genomic infrastructure, particularly local sequencing facilities, is essential to ensure that genomic research for clinical applications can be effectively conducted. Additionally, research institutions with established sequencing infrastructure can partner with healthcare facilities to support the use of NGS in diagnostic and genetic screening services. To fulfil this, there is also the need to train genomic practitioners in NGS, and bioinformaticians for analysis of the genetic data. However, retaining these skilled scientists within the healthcare system is equally important, requiring strong local government support and targeted investment to sustain and expand this expertise in the health sector.

To improve understanding of the disease, there is a need to expand research efforts and studies focused on African populations (65). Such data will inform the development of diagnostic and genetic tools capable of detecting pathogenic variants in patients with DCM. This will enable the accurate integration of population-specific genomic information into routine clinical practice, which is essential for improving disease management and reducing the prolonged diagnostic journey often experienced by patients with idiopathic DCM. While enhancing local capacity remains a crucial endeavour, leveraging the benefits of international partnerships is equally significant for the progression of genomic research and its application within healthcare systems. Collaborative efforts between researchers from Africa and global consortia have produced favourable outcomes, particularly in findings related to the genetic framework of African populations.

## 7 Conclusion

The clinical utility of NGS in elucidating the genetic diversity of DCM in African populations is increasingly evident, with important implications for diagnosis, risk stratification, and clinical management. Despite evidence that DCM in African populations often presents at a younger age and with greater severity, the implementation and integration of NGS into routine clinical care across the continent remains limited. This suboptimal uptake is driven by multiple barriers, including limited access to sequencing infrastructure, high costs, shortages of trained genomic and bioinformatics personnel, inadequate clinical genetic services, and the underrepresentation of African populations in global reference databases. This review highlights these challenges and outlines potential strategies to address them, such as capacity building, regional collaborations, investment in infrastructure, and context-appropriate implementation frameworks.

Importantly, there is an urgent need to expand genomic research across Africa, strengthen local expertise and infrastructure, and ensure the inclusion of African genomic data in global genomics initiatives. Such efforts are essential to improve variant interpretation, reduce health disparities, and promote equitable translation of genomic medicine for individuals with DCM in Africa.
